# A Novel Homozygous ITGA2B Variant Associated With Recurrent Epistaxis in a Four-Year-Old Girl: A Case Report

**DOI:** 10.7759/cureus.102900

**Published:** 2026-02-03

**Authors:** Badriah G Alasmari, Shady Wafa, Bandar Alsharidi, Ayman Abualama, Lina Elzubair

**Affiliations:** 1 Pediatrics, Armed Forces Hospital Southern Region, Khamis Mushayt, SAU; 2 Pediatrics, Armed Forces Hospital, Southern Region, Khamis Mushayt, SAU; 3 Pathology, Armed Forces Hospital, Southern Region, Khamis Mushayt, SAU

**Keywords:** bleeding disorders, glanzmann thrombasthenia (gt), nasal epistaxis, von willebrand diseases, whole-exome sequencing

## Abstract

Glanzmann thrombasthenia (GT) is a rare, autosomal recessive platelet aggregation disorder caused by mutations in the ITGA2B and ITGB3 genes. These mutations result in quantitative or qualitative deficiencies in the alpha IIb beta 3 integrin complex, impairing platelet aggregation and leading to recurrent mucocutaneous bleeding. The key findings include absent or severely reduced platelet aggregation with adenosine diphosphate (ADP), epinephrine, and collagen, while aggregation with ristocetin remains normal. While hundreds of pathogenic variants have been identified, the genetic landscape of GT continues to expand with the discovery of novel mutations. GT treatment focuses on controlling bleeding. Hemopoietic stem cell transplantation (HSCT) offers a curative option for severe cases, while gene therapy is a promising future approach. Here, we report the case of a four-year-old female presenting with recurrent bilateral epistaxis and a significant family history of bleeding diathesis. Whole-exome sequencing (WES) revealed a novel homozygous missense variant in ITGA2B (c.655G>T; p.Gly219Cys). This variant, which is absent from public genomic databases, was predicted to be deleterious by multiple in-silico computational tools, supporting a diagnosis of GT. This case identifies a previously unreported pathogenic variant and underscores the critical role of genetic testing in diagnosing unexplained mucocutaneous bleeding, particularly in cases involving consanguinity.

## Introduction

Glanzmann thrombasthenia (GT) is a rare, autosomal recessive platelet-function disorder caused by quantitative or qualitative defects of the platelet alpha IIb beta 3 (GPIIb/IIIa) glycoprotein receptor [1]. This defect impairs the final common pathway of platelet aggregation, leading to a lifelong predisposition to mucocutaneous bleeding episodes [2]. While the worldwide prevalence of GT is estimated at approximately 1:1,000,000, it is significantly higher in Saudi Arabia, where it is estimated at 1:10,000 in specific regions such as Al Madinah, a phenomenon largely attributed to high rates of consanguinity [3]. The genes encoding these vital integrins, ITGA2B and ITGB3, are located on chromosome 17q21. Mutations in either of these genes can result in the GT phenotype, and to date, hundreds of distinct variants have been reported globally [4]. Clinical manifestation typically requires the patient to be either homozygous for a specific mutation or a compound heterozygote for two different mutations [5]. Despite the extensive catalog of known mutations, novel variants continue to be discovered, reflecting an ongoing expansion of the molecular spectrum of the disease. In a recent multi-center clinical study, the most frequent presenting symptom of GT was epistaxis (39.1%), followed by cutaneous bleeding (23%), gastrointestinal bleeding (19.5%), gingival bleeding (10.3%), and post-circumcision bleeding (5.7%), while a small minority (1.1%) presented initially with intracranial hemorrhage [6]. Laboratory diagnosis typically reveals a normal platelet count and normal prothrombin time (PT) and activated partial thromboplastin time (aPTT), but is marked by a prolonged bleeding time and a characteristic lack of platelet aggregation in response to physiological agonists such as adenosine diphosphate (ADP), collagen, and epinephrine. Notably, platelet adhesion in response to ristocetin remains preserved, and flow cytometry typically confirms defective expression of the GPIIb-IIIa glycoproteins [7].

Here, we report the case of a four-year-old girl presenting with recurrent mucocutaneous bleeding who was found to harbor a novel homozygous missense variant of ITGA2B (c.655G>T; p.Gly219Cys). This case serves to further expand the known mutational spectrum associated with inherited platelet function disorders

## Case presentation

**Figure 1:**
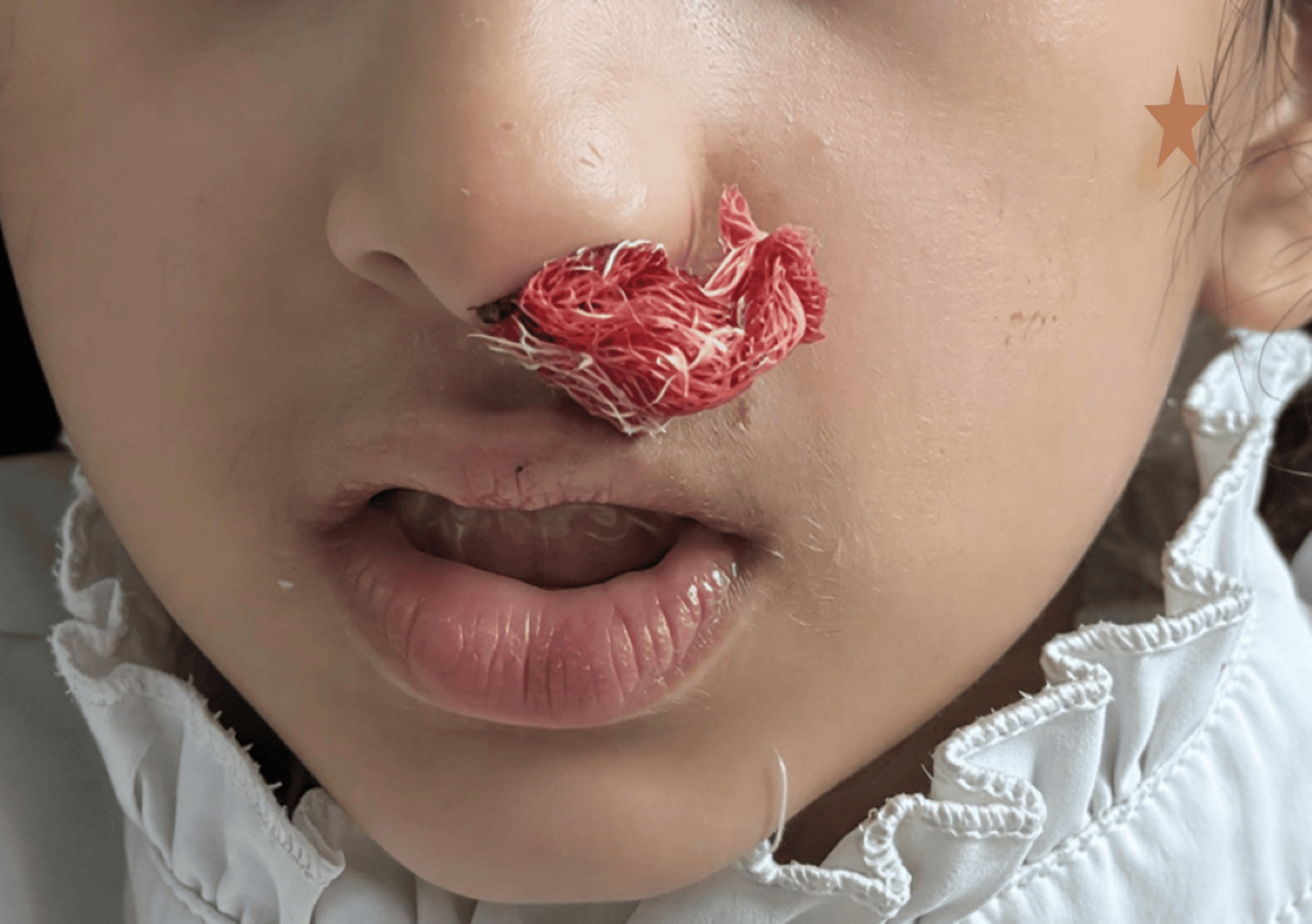
Active mucocutaneous bleeding from the nose with oral pallor.

**Figure 2:**
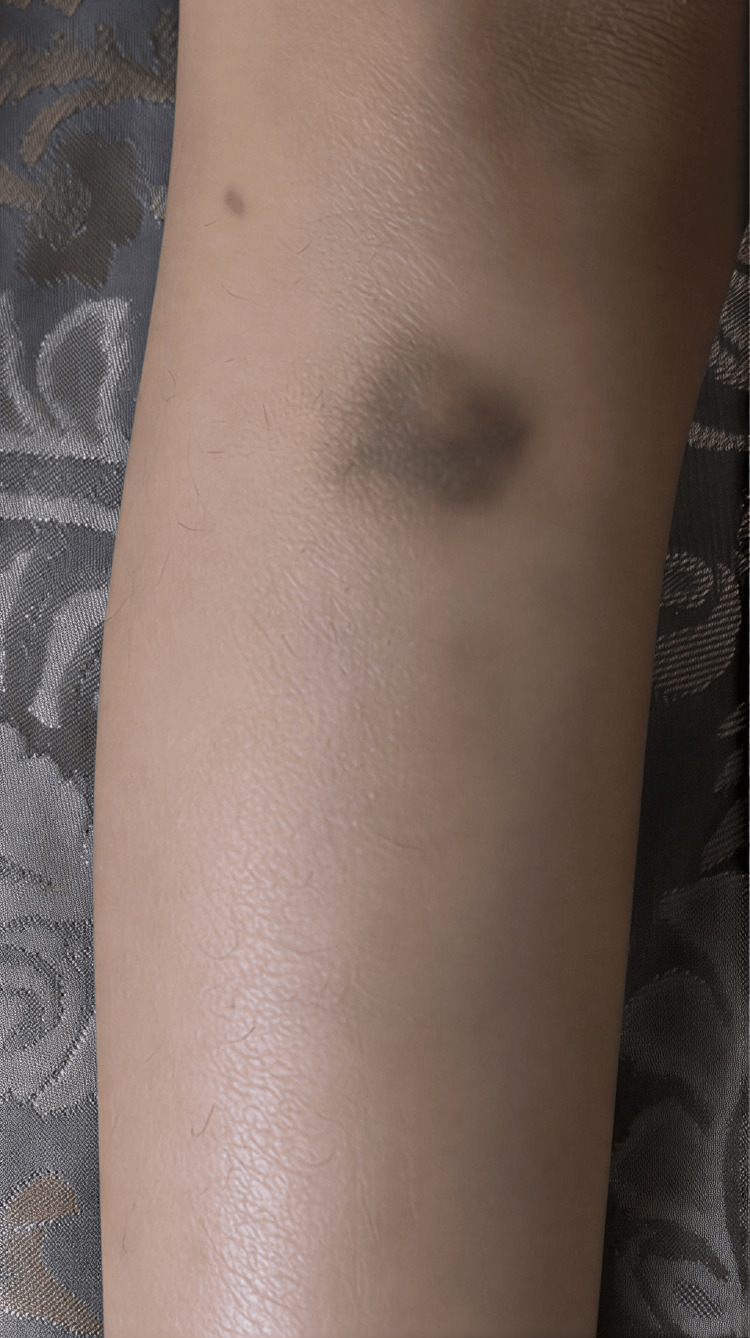
Spontaneous ecchymosis on the lower limb, with bluish discoloration indicating subcutaneous bleeding.

A four-year-old Saudi girl was born at a local hospital at full term via emergency cesarean section for fetal distress to consanguineous parents. There was a positive family history of bleeding disorders, including menorrhagia in female relatives and occasional epistaxis among cousins. The patient’s parents are first cousins, suggesting possible autosomal recessive inheritance. Soon after birth, she was found to have epistaxis, dark stool, multiple skin bruises all over her body, and a low hemoglobin level (10 g/dL), requiring neonatal intensive care unit (NICU) admission for evaluation and blood transfusion. There was no evidence of acute hemolysis or infection, and the initial bleeding workup showed mild coagulopathy with a normal platelet count. Her condition gradually improved after she received a single transfusion of packed red blood cells (PRBCs) and two doses of fresh frozen plasma (FFP). She was discharged home after two weeks in a stable condition with follow-up instructions. The parents did not follow up regularly with their local hospital. Later, they noticed that the patient had recurrent mild epistaxis, dark stool with scattered skin bruises not preceded by trauma, and otherwise the patient remained in stable condition. At 18 months of age, she required another PRBC transfusion at her local hospital for symptomatic anemia secondary to moderate epistaxis. She also underwent nasal packing and was subsequently discharged in stable condition with iron supplementation, as there was evidence of iron deficiency anemia. Despite her recurrent bleeding tendency, the patient was not fully investigated for bleeding disorders until her first admission to our hospital at age 3, when she presented with spontaneous moderate-to-severe bilateral nasal bleeding (Figure 1), bruises all over her body (Figure 2), pallor, and tachycardia. Her hemoglobin level was 6.5 g/dL, with laboratory evidence of iron deficiency anemia. There was no jaundice, lymphadenopathy, or organomegaly, and the remainder of her clinical examination was unremarkable. Her coagulation profile and platelet count were normal; therefore, further bleeding workup, including platelet function assay (PFA), coagulation factor XIII, and von Willebrand factor (VWF) assay, was sent (Table 1). She was supported with PRBC transfusion, tranexamic acid, and nasal packing. The bleeding gradually stopped over two days, and she was discharged in good condition with iron supplementation and follow-up at the hematology clinic. Later, her bleeding workup revealed low VWF, normal factor XIII, and no result for PFA due to unavailability of the reagent. She was diagnosed with type 1 VWD, and the family was counseled and scheduled for regular hematology clinic follow-up. During subsequent follow-up, the patient was noted to have recurrent mild-to-moderate spontaneous nasal bleeding and easy skin bruising, and she required two further admissions over six months for moderate-to-severe bilateral epistaxis and gum bleeding. These episodes were managed with combined VWF/FVIII injections, tranexamic acid therapy, and nasal packing. Due to the recurrence of moderate-to-severe mucocutaneous bleeding, the diagnosis of type 1 VWD was questioned, and further investigations, including whole exome sequencing (WES) and repeated VWF assays, were ordered. Platelet flow cytometry was not available and therefore was not performed. The repeated VWF assay was normal, and WES revealed a homozygous missense variant in the ITGA2B gene, c.655G>T (p.Gly219Cys). This variant affects a highly conserved amino acid within the ITGA2B protein, which plays a critical role in platelet αIIbβ3 integrin function, essential for platelet aggregation, and is consistent with a diagnosis of GT. The parents were re-counseled and offered segregation testing, but they declined. Currently, the patient has regular follow-up with hematology and ENT clinics, receives iron supplementation and tranexamic acid as needed, and maintains a hemoglobin level in the range of 9-10 g/dL.

**Table 1:** Laboratory findings at the time of admission at age three years. WBC, white blood cell count; HCT, hematocrit; MCV, mean corpuscular volume; RDW, red cell distribution width; TIBC, total iron binding capacity; UIBC, unsaturated iron binding capacity; vWF:Ac, Von Willebrand factor activity

Description	Result	Unit	Reference range
WBC	7.41	10^9^/L	4.5-13.5
RBC	2.41	10^12^/L	4.1-5.3
Hemoglobin	6.3	g/dL	10.9-15
HCT	29.75	%	34-44
MCV	80.3	fL	76-96
RDW	19.8	%	11-14
Platelets	236	10^9/L	150-450
Reticulocyte	5.82	%	0.5-2.5
Serum iron	4.8	µmol/L	10.7-32.2
TIBC	63.0	µmol/L	45-79
UIBC	58.2	µmol/L	27.7-63.6
Transferrin saturation	7.62	%	<50%
vWF:Ac (Initial)	42.3	%	49.5-187
vWF:Ac (Repeated)	97	%	49.5-187

## Discussion

GT is a rare, autosomal recessive bleeding disorder characterized by a quantitative or qualitative defect in the αIIbβ3 integrin. This complex serves as the primary receptor for fibrinogen; its dysfunction prevents platelet aggregation, leading to severe mucocutaneous bleeding despite a normal platelet count [8]. This case is particularly significant as it describes a novel homozygous variant (c.655G>T; p.Gly219Cys) in a four-year-old girl, highlighting the diagnostic challenges in distinguishing GT from other more common coagulopathies like VWD. The initial diagnosis of VWD type 1 in our patient underscores a common clinical pitfall. In pediatric patients presenting with mucocutaneous bleeding and a normal platelet count, VWD is often the first suspicion. Our patient’s initial low VWF levels likely represented a *false positive* or transiently low level, which can occur due to blood group (type O), stress, or testing variability [5]. However, the failure of VWF/Factor VIII concentrates to control the recurrent epistaxis was a critical clinical *red flag*. In GT, unlike VWD, the bleeding stems from the inability of platelets to form a primary plug. This case demonstrates that when a patient with a *mild* diagnosis like VWD type 1 presents with severe, transfusion-dependent bleeding, clinicians must look toward rarer platelet function disorders. The ITGA2B gene encodes the αIIb subunit of the integrin receptor. The p.Gly219Cys variant identified in this patient is located in a highly conserved region of the protein. Glycine, being the smallest amino acid, often provides necessary flexibility for protein folding. Replacing it with cysteine, a bulky amino acid capable of forming aberrant disulfide bonds, likely disrupts the structural integrity of the αIIb subunit or its ability to complex with the β3 subunit [8]. To our knowledge, this specific c.655G>T substitution has not been previously characterized in major genomic databases, making it a novel contribution to the mutational landscape of GT. The management of GT remains challenging, primarily relying on local measures, antifibrinolytics like tranexamic acid, and iron replacement for chronic anemia [1]. While platelet transfusions are the gold standard for severe bleeding, they carry the risk of alloimmunization, which can render future transfusions ineffective. In this case, the lack of specialized PFAs (PFA-100) and flow cytometry in the local hospital setting delayed the diagnosis. This emphasizes the vital role of whole exome sequencing (WES) as a diagnostic tool.

## Conclusions

This case adds a novel ITGA2B variant to the global registry of GT mutations. Patients usually present with easy bruising and bleeding from epistaxis and dental extractions. It serves as a reminder that a normal platelet count does not rule out a severe platelet disorder. Clinicians should maintain a high index of suspicion for GT in cases of severe mucocutaneous bleeding refractory to standard VWD treatments, particularly in the context of parental consanguinity.
